# Human Herpesvirus 8 Seroprevalence, China

**DOI:** 10.3201/eid1801.102070

**Published:** 2012-01

**Authors:** Tiejun Zhang, Xiaodan Shao, Yue Chen, Tao Zhang, Veenu Minhas, Charles Wood, Na He

**Affiliations:** Fudan University, Shanghai, People’s Republic of China (T. Zhang, X. Shao, Tao Zhang, V. Minhas, C. Wood, N. He);; University of Nebraska-Lincoln, Lincoln, Nebraska, USA (T. Zhang, V. Minhas, C. Wood);; University of Ottawa, Ottawa, Ontario, Canada (Y. Chen)

**Keywords:** HIV, HHV-8, herpesvirus 8, viruses, prevalence, systematic review, China

## Abstract

To summarize the seroprevalence of human herpesvirus 8 (HHV-8) in mainland China, we conducted a systematic review and meta-analysis based on available literature. Data show that differences in HHV-8 prevalence vary considerably among different ethnic groups and geographic regions. Blood-borne transmission could be a potential route for HHV-8 infection in China.

Human herpesvirus 8 (HHV-8) is the infectious etiologic agent associated with Kaposi sarcoma, primary effusion lymphoma, and multicentric Castleman disease. Worldwide seroprevalence of HHV-8 varies: generally low to moderate for populations in Western countries and Asia (*1*–*4*) but as high as 50% for the general population in sub-Saharan Africa and higher for HIV-positive populations (*5*–*7*). The transmission modes of HHV-8 may also differ in different geographic areas and subpopulations; sexual and nonsexual transmission have been described (*8*–*10*). Blood-borne transmission may exist, especially among intravenous drug users (IVDUs) and blood recipients (*11*).

The Ministry of Health of China, the United Nations Program on HIV/AIDS, and the World Health Organization estimate that ≈320,000 HIV/AIDS cases have been reported in China (*12*). However, the epidemiologic characteristics of HHV-8 infection, a severe HIV/AIDS opportunistic infection, have not been well described for China. Therefore, we conducted a systematic review and metaanalysis on the basis of available data for HHV-8 epidemiology from mainland China to have a better understanding of the prevalence, variation, and factors associated with its transmission.

## The Study

A comprehensive literature search of published studies indexed in global and databases in China during 1995–2010 was conducted. Initially, 125 reports published in English and 223 in Chinese concerning the seroprevalence in mainland China were identified. Among them, 85 articles published in England and 178 articles published in China were excluded after title and abstract screening. After reading the full text, we excluded another 33 English and 26 Chinese articles. Finally, 26 publications were included in this systematic review and have been summarized in Technical Appendix Table 1. These studies were cross-sectional and were conducted in 8 of the 34 provinces. A substantial number (35.5%) of these studies were conducted in the Xinjiang Uygur Autonomous Region. Most samples tested were serum or plasma with few exceptions (1 whole blood, 1 peripheral blood mononuclear cells); sample sizes ranged from 37 to 4,461 (median 242, interquartile range 199–520). Overall, 18,547 participants were involved in the present analysis, and among them 15,913 were from the general population, 1,970 were immunocompromised patients, and 664 were IVDUs. Laboratory methods for all included studies were reported (19 detected HHV-8 by ELISA, 3 by PCR, and 4 by immunofluorescent assay.

The prevalence of HHV-8 pooled from reviewed studies was 11.3% (95% CI 7.2–15.5) for the general population, 22.2% (95% CI 12.7–31.8) for immunocompromised patients, and 31.2% (95% CI 27.7–34.7) for IVDUs. The prevalence among the general population was found to be the lowest in Guangdong Province and the highest in Xinjiang Province. A similar regional variation was found for immunocompromised persons. Among IVDUs, the prevalence was 34.3% (95% CI 28.3–40.3) in Zhejiang and 29.6% (95% CI 25.3–33.9) in Xinjiang Uygur Autonomous Region (Technical Appendix Table 2; Figure).

**Figure:**
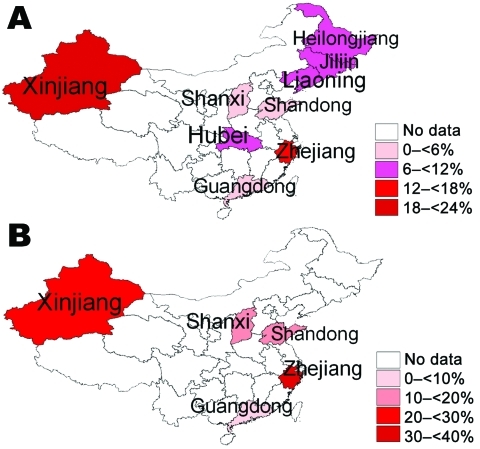
Regional distribution of pooled human herpesvirus 8 (HHV-8) prevalence in A) the general population and B) immunocompromised patients, China.

Five studies, including 4,637 persons of Han ethnicity and 4,011 persons of ethnic minorities (2,040 Uygur, 1,169 Kazak, 200 Khalkas, 173 Hue, and 429 other) conducted in the Xinjiang Uygur Autonomous Region were analyzed for association of ethnicity with HHV-8 prevalence (Technical Appendix Figure, panel A). The risk was significantly lower for the Han group than for other ethnic groups (odds ratio [OR] = 0.59, 95% CI 0.55–0.76). For the Han group, the pooled prevalence of HHV-8 in Xinjiang Uygur Autonomous Region was significantly higher when compared with that for other regions, 14.4% (95% CI 9.0–19.8) versus 6.4% (95% CI 4.1–8.6). Ten combined studies, with 5,716 male and 4,708 female participants, respectively, were included in meta-analysis of association between sex and HHV-8 infection (Technical Appendix Figure, panel B). There was no significant difference between the sexes: pooled OR 0.94 (95% CI 0.84–1.04).

Seven studies, with 863 HIV-positive patients and 3,438 negative controls, were included in the analysis. All studies yielded a significant difference in HHV-8 infection between HIV-positive and HIV-negative participants; ORs for individual studies ranged from 1.50 to 4.27, and the pooled OR was 2.97 (95% CI 2.22–3.97) (Technical Appendix Figure, panel C). However, a significant publication bias was detected (Egger test p = 0.013; Begg test p = 0.016). A visual inspection of the funnel plot suggested that some large or small studies with negative or null results were not published (data not shown).

Few studies were designed to address the issue of possible transmission routes among the population of china. Six studies had information on possible blood transmission. Two blood transfusion studies and 4 studies of IVDUs included 837 persons who reported having been exposed to blood contact i.e., needle sharing and 1,397 who were never exposed (Technical Appendix Figure, panel D). Substantial heterogeneity (I^2^ 87%, p<0.001, by test for heterogeneity) was detected among those studies; therefore, a random-effects model was used to estimate the OR. No publication bias was detected (Begg test p = 0.707; Egger test p = 0.363). OR showed a marginal association of HHV-8 prevalence with blood transfusion (OR 2.01, 95% CI 0.89–4.56) for possible blood transmission.

## Conclusions

This systematic review indicated that HHV-8 prevalence in China varies in different regions. Pooling of data from 26 studies provided us with a large sample size, which is one of the strengths of the study. Also, we included studies that were published in the Chinese language and were not accessible to the international community. The results of this meta-analysis show that HHV-8 prevalence was higher in the Xinjiang Uygur Autonomous Region than other areas in general and among high-risk populations. Historically, Xinjiang Uygur Autonomous Region has been regarded as an area in which Kaposi sarcoma is endemic (*13*). Notably, geographic variations of HHV-8 infection within China are not well known and need to be investigated as well.

It has been well documented that HHV-8 prevalence is higher among HIV-infected persons (*14*,*15*). In mainland China, we found a 3-fold increase in HHV-8 infection among persons with HIV compared with HIV-noninfected persons. Given the rapid increase of HIV/AIDS cases in China, HHV-8 could become a severe public health issue in the future.

According to data from the Xinjiang Uygur Autonomous Region, minority groups were at higher risk for HHV-8 infection than the Han ethnic group. Although there was evidence for considerable heterogeneity among the studies, the association between ethnicity and HHV-8 risk showed that minorities were at higher risk for HHV-8 infection when compared with the Han ethnic group. The reasons behind this association are not well elucidated. Because all of the comparisons of HHV-8 difference between minority groups and the Han ethnic group are from the Xinjiang Uygur Autonomous Region, epidemiologic confirmation of this observation would require data from other regions, which is currently unavailable. Our analysis showed a marginally significant association between blood contact and HHV-8 infection; heterogeneity among studies was substantial. These data indicate that blood-borne transmission could occur among the Chinese population, a finding that is consistent with previous reports from other countries (*11*).

This study has some limitations. The studies included in this meta-analysis were not evenly distributed throughout China because information was not available from all the regions. Also, all of the studies might have used different methods for HHV-8 detection because of the lack of a standard assay; prevalence estimates may have been underestimated.

In summary, this meta-analysis clearly shows that the distribution of HHV-8 seroprevalence varies in China. The available information is still too limited to fully understand HHV-8 prevalence and the risk factors associated with transmission. Further studies are urgently needed to explore the epidemiology of HHV-8 infection in different subpopulations in China.

## Supplementary Material

Technical Appendix Meta-analysis of herpesvirus 8 (HHV8) prevalence among subgroups in China.
