# Supplementary material for: Human Herpesvirus 8 Seroprevalence, China
Source: Emerg Infect Dis. 2012 Jan;18(1):150–2. doi: 10.3201/eid1801.102070 (PMC3310085; doi:10.3201/eid1801.102070)
Supplement: Technical Appendix — Meta-analysis of herpesvirus 8 (HHV8) prevalence among subgroups in China. [file 10-2070-Techapp_6p.pdf]

Article DOI: http://dx.doi.org/10.3201/eid1801.102070

Human Herpesvirus 8 Seroprevalence, China

Technical Appendix

Technical Appendix Table 1. Summary of data from literature on HHV8 prevalence, China*

Author, year	District	Design	Sample	Detection method	Sample collection year(s)	Target population	Sample size	HHV-8 prevalence (95% CI)
Qi et al., 2005	Shandong	Cross-sectional	Serum	ELISA	2003/2004	General population	303	6.3 (3.6–9.0)
Qi et al., 2006	Shandong	Cross-sectional	Serum	ELISA	2003/2004	General population	520	6.0 (4.0–8.0)
Qi et al., 2007	Shandong	Cross-sectional	Serum	ELISA	NA	General population	230	5.7 (2.7–8.7)
	Shandong	Cross-sectional	Serum	ELISA	NA	Immunocompromised persons†	86	16.3 (8.5–24.1)
Li, et al, 2008	Shandong	Cross-sectional	Plasma	ELISA	2005/2006	General population	460	5.7 (3.6–7.8)
Wang et al., 2000	Zhejiang	Cross-sectional	Serum/ plasma	ELISA	1998/1999	Drug user	242	34.3 (28.3–40.3)
Wang et al., 2001	Zhejiang	Cross-sectional	Plasma	ELISA	1998/1999	General population	241	16.2 (11.5–20.9)
Zhu et al., 2008	Zhejiang	Cross-sectional	Plasma	ELISA	NA	Immunocompromised persons	214	36.4 (30.0–42.8)
			Plasma	ELISA	NA	General population	122	13.0 (7.0–19.0)
Fu et al., 2006‡	Guangdong	Cross-sectional	Whole blood	PCR	NA	General population	239	0.8 (0.0–1.9)
Fu et al., 2006‡	Guangdong	Cross-sectional	Plasma	ELISA	2004/2005	General population	3,135	0.2 (0.0–0.4)
Guan et al., 2008	Guangdong	Cross-sectional	Serum	ELISA	NA	Immunocompromised persons	167	0.6 (0.0–1.8)
Fu et al., 2009‡	Hubei	Cross-sectional	Serum	ELISA	2004/2005	General population	560	6.8 (4.7–8.9)
Fang et al., 2006	Hubei	Cross-sectional	Serum	ELISA	2004/2005	General population	560	5.2 (3.4–7.0)
Wang et al., 2002	Dongbei§	Cross-sectional	PMBC	PCR	NA	General population	230	7.8 (4.3–11.3)
Zhang et al., 2010	Shanxi	Cross-sectional	Plasma	IFA	2004/2005	General population	315	4.8 (2.4–7.2)
			Plasma	IFA	2004/2005	Immunocompromised persons	305	15.4 (11.3–19.5)
Du et al., 2000	Xinjiang	Cross-sectional	Serum	IFA	NA	General population	1,071	24.5 (21.9–27.1)
Sun, et al., 2002	Xinjiang	Cross-sectional	Serum	ELISA	1998–2000	Immunocompromised persons	56	28.5 (16.7–40.3)
Sun et al., 2003	Xinjiang	Cross-sectional	Serum	IFA	2000	General population	406	15.5 (12.0–19.0)
Qing et al., 2005	Xingjang	Cross-sectional	Serum	PCR	2002/2003	General population	68	22.1 (12.2–32.0)
Wu et al., 2005	Xinjiang	Cross-sectional	Serum	IFA	NA	General population	204	30.4 (24.1–36.7)
He et al., 2007‡	Xinjiang	Cross-sectional	Serum	ELISA	NA	Immunocompromised persons	155	26.5 (19.6–33.4)
He et al., 2007‡	Xinjiang	Cross-sectional	Serum	ELISA	2004	Immunocompromised persons	482	25.3 (21.4–29.2)
Fu et al., 2009‡	Xinjiang	Cross-sectional	Serum	ELISA	2007	General population	2,228	19.2 (17.6–20.8)

Author, year	District	Design	Sample	Detection method	Sample collection year(s)	Target population	Sample size	HHV-8 prevalence (95% CI)
			Serum	ELISA	2007	Immunocompromised persons	37	43.2 (27.2–59.2)
	Hubei	Cross-sectional	Serum	ELISA	2007	General population	560	9.5 (7.1–11.9)
Yang et al., 2009	Xinjiang	Cross-sectional	Serum	ELISA	2006	Immunocompromised persons	468	14.3 (11.1–17.5)
Wang, et al., 2010	Xinjiang	Cross-sectional	Serum	ELISA	NA	General population	4,461	20.4 (19.2–21.6)
Yang et al., 2010‡	Xinjiang	Cross-sectional	Serum	ELISA	2006	Drug user	223	28.7 (22.8–34.6)
Yang et al., 2010‡	Xinjiang	Cross-sectional	Serum	ELISA	2006	Drug user	199	30.6 (24.2–37.0)

*HHV8, human herpesvirus 8; NA, not applicable.
†Immunocompromised persons include patients with tumors, organ transplant recipients, HIV-positive patients, and other patients with immunosuppressed-related conditions.
‡From same first author in 1 year.
§Dongbei includes the provinces of Heilongjiang, Jilin, and Liaonin.

Technical Appendix Table 2. Distribution of HHV-8 prevalence among different subpopulations in mainland China*

Population type and province	Author, year	Sample size	HHV-8 prevalence (95% CI)	Pooled prevalence by region (95% CI)
General population				
Shandong	Qi et al., 2005	303	6.3 (3.6–-9.0)	5.9 (4.7–7.1)
	Qi et al., 2006	520	6.0 (4.0–8.0)	
	Qi et al., 2007	230	5.7 (2.7–8.7)	
	Li et al., 2008	460	5.7 (3.6–7.8)	
Zhejiang	Wang et al., 2001	241	16.2 (11.5–20.9)	15.0 (11.3–18.7)
	Zhu et al., 2008	122	13.0 (7.0–19.0)	
Guangdong	Fu et al., 2006a	239	0.8 (0.0–1.9)	0.2 (0.0–0.5)
	Fu et al., 2006b	3,135	0.2 (0.0–0.4)	
Hubei	Fu et al., 2009a	560	6.8 (4.7–8.9)	7.1 (4.7–9.4)
	Fu et al., 2009b	560	9.5 (7.1–11.9)	
	Fang et al., 2006	560	5.2 (3.4–7.0)	
Dongbei	Wang et al., 2002	230	7.8 (4.3–11.3)	7.8 (4.3–11.3)
Shanxi	Zhang et al., 2010	315	4.8 (2.4–7.2)	4.8 (2.4–7.2)
Xinjiang	Du et al., 2000	1,071	24.5 (21.9–27.1)	21.2 (18.6–23.9)
	Sun et al., 2003	406	15.5 (12.0–19.0)	
	Qing et al., 2005	68	22.1 (12.2–32.0)	
	Wu et al., 2005	204	30.4 (24.1–36.7)	
	Fu et al., 2009b	2,228	19.2 (17.6–20.8)	
	Wang et al., 2010	4,461	20.4 (19.2–21.6)	
Pooled	NA	NA	11.3 (7.2–15.5)	NA
Immunocompromised persons				
Shandong	Qi et al., 2007	86	16.3 (8.5–24.1)	16.3 (8.5–24.1)
Zhejiang	Zhu et al., 2008	214	36.4 (30.0–42.8)	36.4 (30.0–42.8)
Guangdong	Guan et al., 2008	167	0.6 (0.0–1.8)	0.6 (0.0–1.8)
Shanxi	Zhang et al., 2010	305	15.4 (11.3–19.5)	15.4 (11.3–19.5)
Xinjiang	Sun et al. 2002	56	28.5 (16.7–40.3)	25.6 (17.7–33.4)
	He et al., 2007a	155	26.5 (19.6–33.4)	
	He et al., 2007b	482	25.3 (21.4–29.2)	
	Fu, t al., 2009b	37	43.2 (27.2–59.2)	
	Yang et al., 2009	468	14.3 (11.1–17.5)	
Pooled	NA	NA	22.2 (12.7–31.8)	NA
IV drug users				
Zhejiang	Wang et al., 2000	242	34.3 (28.3–40.3)	34.3 (28.3–40.3)
Xinjiang	Yang et al., 2010a	223	28.7 (22.8–34.6)	29.6 (25.2–33.9)
	Yang et al., 2010b	199	30.6 (24.2–37.0)	
Pooled	NA	NA	31.2 (27.7–34.7)	NA

*HHV-8, human herpesvirus 8; a, b: From the same first author in one year; IV, intravenous. NA, not applicable.

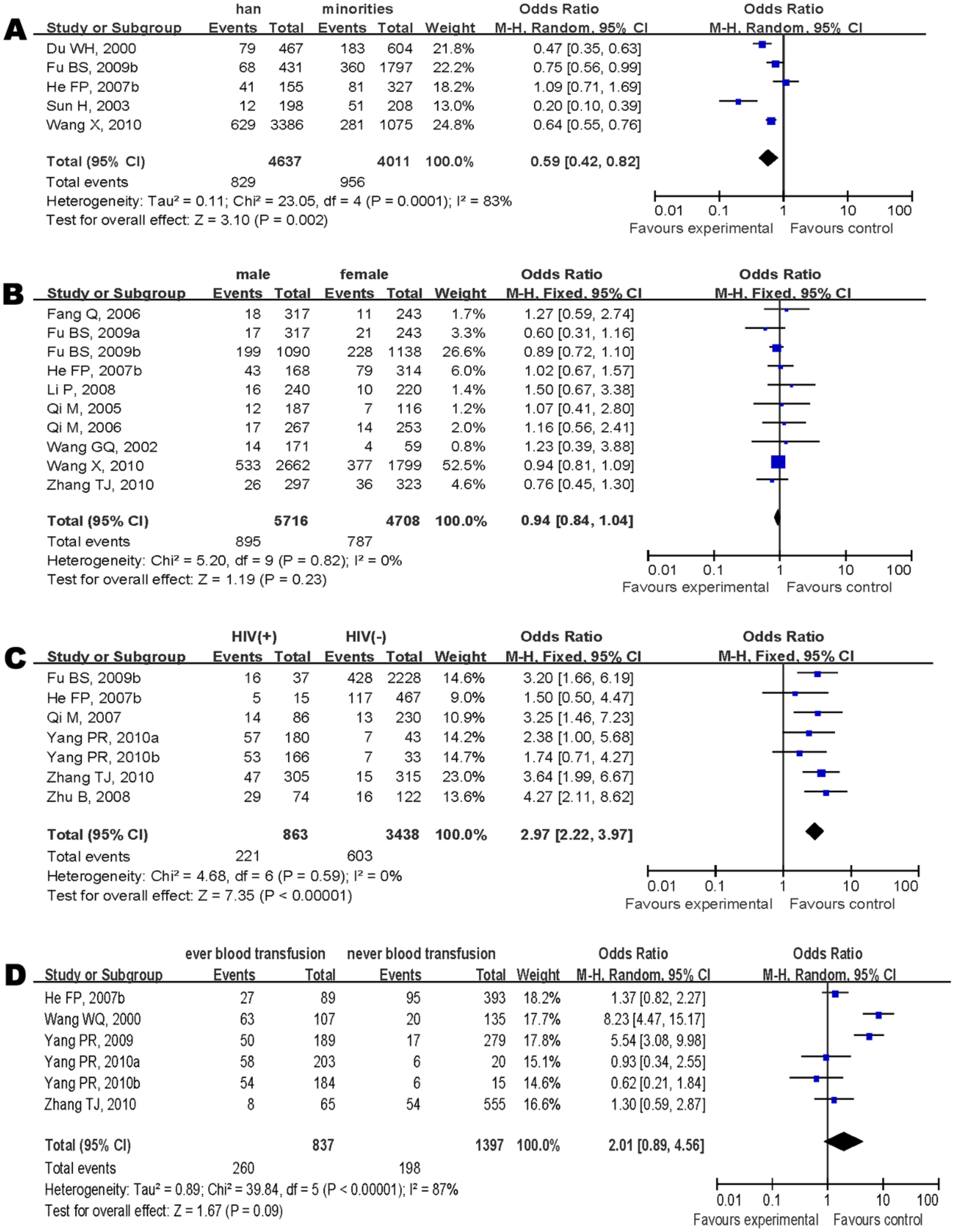

Technical Appendix Figure. Meta-analysis of herpesvirus 8 (HHV8) prevalence among subgroups in China. A) Comparison of (HHV-8 prevalence between persons of Han ethnicity and of minority ethnicity in Xinjiang, China; B) Comparison of HHV-8 prevalence between male and female study participants in China; C) Comparison of HHV-8 prevalence between persons

with and without HIV infection in China; and D) Comparison of HHV-8 prevalence between blood contact and noncontact subjects.

Bibliography

Du W, Chen G, et al. Antibody to human herpesvirus type-8 in the general populations of Xinjiang Autonomous Region (A.R.). Zhonghua Shi Yan He Lin Chuang Bing Du Xue Za Zhi. 2000;14:44–6. PubMed

Fang Q, Liu J, Bai Z, Kang K, He Z, Hu Z, et al. Seroprevalence of Kaposi's Sarcoma-associated Herpesvirus in the General Population from Hubei Province. Virol Sin. 2006;21:97–101.

Fu B, Li B, Ouyang X, Zeng Y, Xu F, Wang L. Expression of Kaposi s Sarcoma-associated Herpesvirus ORFK8.1 and Its Preliminary Diagnostic Application. Virol Sin. 2009;24:202–8. http://dx.doi.org/10.1007/s12250-009-3029-0

Fu B, Sun F, Li B, Yang L, Zeng Y, Sun X, et al. Seroprevalence of Kaposi's sarcoma-associated herpesvirus and risk factors in Xinjiang, China. J Med Virol. 2009;81:1422–31. PubMed http://dx.doi.org/10.1002/jmv.21550

Fu Y, Zheng Y, Guang H, Wang C, Jiang C. Seroepidemiology of human herpesvirus 8 among unpaid blood donors in Guangzhou. Mod Prev Med. 2006;33:188–9.

Fu Y, Zhou H, Guang H, Nie Y, Wang C, Jiang C. Detection and sequence analysis of human herpesvirus 8 DNA in unpaid blood donors in Guangzhou. J Trop Med. 2006;6:376–8.

Guan H, Fu Y, Zhou Z, Zhou H. HHV8 antibody test among blood recipients and HBV positive cases. Chin J Blood Tansfusion. 2008;21:692–4.

He F, Wang X, He B, Feng Z, Lu X, Zhang Y, et al. Human herpesvirus 8: serovprevalence and correlates in tumor patients from Xinjiang, China. J Med Virol. 2007;79:161–6. PubMed http://dx.doi.org/10.1002/jmv.20730

He, F., X. Wang, Zhang Y, Hui Y, Lin R, Lu X et al. The epidemiological study on Kaposi's sarcoma asciociated herpasverus among Han patients in Xinjiang. Journal of Xinjiang Medical University. 2007;103-6.

Mei Q, Zhao W, Zhou Y, Luan Y, Bian J, Wang H, et al. Prevalance of human herpesvirus 8(HHV-8)IgG and its associated risk factors in blood donors from Jinan region. [Health Sciences]. Journal of Shandong University. 2006;44:328–31.

Mei Q, Ming ZW, Ping YX, Hui JJ, Bin ZY, Hong W, et al. HHV-8 seroprevalence in blood donors and HIV-positive individuals in Shandong area, China. J Infect. 2007;55:89–90. PubMed http://dx.doi.org/10.1016/j.jinf.2006.10.046

Mei Q, Ming ZW, Zhang X, Zhou Y, Luan Y, Yu X, et al. Seroepidemiology study on human herpesvirus 8 among health blood donor in Shandong, China. Zhonghua Liu Xing Bing Xue Za Zhi. 2005;26:883.

Peng L, Jian LF. Detection HHV8 infection among health blood donor with ELISA. Chin J Prim Med Pharm. 2008;15:512.

Qin J, Feng L, Tan X, Guo S, Wang X, Zhang W, et al. A case-control study on risk factors of classic Kaposi s sarcoma in Xinjiang. Zhonghua Liu Xing Bing Xue Za Zhi. 2005;26:673–5. PubMed

Sun H, Chen G, Wang L, Jia E, Zhu L, Du W, et al. Human Herpes virus Type-8 Infection in the Mothers and Their Infants of Wulumuqi and Aletai Region. Chin J of Perinat Med. 2003;6:21–3.

Sun H, Zhou M, Chen G, Du W, Zeng Y. Human herpesvirus 8 IgG antibody test among HIV positive population in Urumuqi. Chinese J Exp Clin Virol. 2002;16:195.

Wang G, Xu H, Zhao Y, Wang Y, Chen H. Detection of Human Herpesvirus 8 in Healthy Blood Donors in Northeast China. Chin J Derm Venereol. 2002;16:83–6.

Wang W, Tong J, Zhao X, Zhang X, Zhang K. Serological study on Kaposi's sarcoma–associated virus among health blood donor in Zhejiang, China. Chin J Blood Tansfusion. 2001;14:46–7.

Wang W, Zhu B, Zhao X, Zhang X, Shen Y, Gao S. Detection of Kaposi's sarcoma–associated virus among illegal drug user. Natl Med J China. 2000;80:597.

Wang X, He B, Zhang Z, Liu T, Wang H, Li X, et al. Human herpesvirus-8 in northwestern China: epidemiology and characterization among blood donors. Virol J. 2010;7:62. PubMed http://dx.doi.org/10.1186/1743-422X-7-62

Wu W, Pu X, Sun H, Xin Y. Detection of human herpesvirus 8 antibody and DNA among Kaposis`s sarcoma patients. China J Dermatol. 2005;38:765.

Yang P, Guo S, Tan X, Yang L, Fu B, Wang L, et al. Seroepidemiology of Kaposi's sarcoma–associated herpesvirus in Uigur male drug users from a place in Xinjiang. [Natural Science]. Journal of Shihezi University. 2010;28:68–71.

Yang, P., X. Tan, Guo S, Yang L, Zeng Y, Fu B, et al. Research of Kaposis's sarcoma–associated herpesvirus in drug users in one city of Xinjiang. Mod Prev Med. 2010;37:107–9.

Yang PR, Guo SX, Tan XH, Yang L. Research on co-infections of HIV and human herpesvirus-8 among the Uygur high-risk groups in a city, Xinjiang. Zhonghua Yu Fang Yi Xue Za Zhi. 2009;43:960–4. PubMed

Zhang T, He N, Ding Y, Crabtree K, Minhas V, Wood C. Prevalence of human herpesvirus 8 (HHV8) and hepatitis C virus (HCV) in a rural community with high risk for blood borne infections in central China. Clin Microbiol Infect. 2011;17:395–401. PubMed http://dx.doi.org/10.1111/j.1469-0691.2010.03287.x

Zhu B, Chen Y, Xie J, Wu N, Shendu J, Wang Y. Kaposi's sarcoma–associated herpesvirus (KSHV) infection: endemic strains and cladograms from immunodeficient patients in China. J Clin Virol. 2008;42:7–12. PubMed http://dx.doi.org/10.1016/j.jcv.2007.11.018
